# Quantifying bias in measuring insecticide-treated bednet use: meta-analysis of self-reported vs objectively measured adherence

**DOI:** 10.7189/jogh.08.010411

**Published:** 2018-06

**Authors:** Paul J Krezanoski, David R Bangsberg, Alexander C Tsai

**Affiliations:** 1 Department of Medicine, Zuckerberg San Francisco General Hospital, University of California, San Francisco, San Francisco, California, USA; 2 Department of Pediatrics, Zuckerberg San Francisco General Hospital, University of California, San Francisco, San Francisco, California, USA; 3 Oregon Health Sciences University – Portland State University School of Public Health, Portland, Oregon, USA; 4 Center for Global Health, Massachusetts General Hospital, Boston, Massachusetts, USA; 5 Harvard Medical School, Boston, Massachusetts, USA; 6 Chester M Pierce, MD Division of Global Psychiatry, Massachusetts General Hospital, Boston, Massachusetts, USA

## Abstract

**Background:**

Insecticide-treated bednets (ITNs) are recommended for use by 3.4 billion people at risk of malaria world-wide. Policy makers rely on measurements of ITN use to optimize malaria prevention efforts. Self-reports are the most common means of assessing ITN use, but self-reports may be biased in a way that reduces their reliability as a proxy for ITN adherence. This meta-analysis compared self-reported and two methods which are more objective measures of ITN use to explore whether self-reports overestimate actual ITN adherence.

**Methods:**

A comprehensive search of electronic databases and hand searching reference lists resulted in screening 2885 records and 202 articles were read in full. Sixteen articles with comparable data were chosen for the meta-analysis. Comparable data was defined as self-reported and objectively measured ITN use (observation of a mounted ITN or surprise visits confirming use) at the same unit of analysis, covering the same time period and same population. A random effects model was used to determine a weighted average risk difference between self-reported and objectively measured ITN use. Additional stratified analyses were conducted to explore study heterogeneity.

**Results:**

Self-reported ITN use is 8 percentage points (95% confidence interval CI: 3 to 13) higher than objectively measured ITN use, representing a 13.6% overestimation relative to the proportion measured as adherent to ITN use by objective measures. Wide variations in the discrepancies between self-reports and objective measures were unable to be explained using stratified analyses of variables including location, year of publication, seasonality and others.

**Conclusions:**

Self-reports overestimate ITN adherence relative to objectively measured ITN use by 13.6% and do so in an unpredictable manner that raises questions about the reliability of using self-reported ITN use alone as a surveillance tool and a guide for making policy decisions.

Despite significant improvements in malaria control over the last 15 years, malaria continues to represent a significant burden for the world’s poorest people [1]. A major focus of malaria prevention efforts has been the distribution of insecticide-treated bednets (ITNs) on a massive scale. ITNs are one of the most powerful and cost-effective tools for malaria prevention [2]. The World Health Organization (WHO) features ITNs prominently as the foundation for vector control [3] and calls for “universal access to and utilization of ITNs” by all 3.2 billion people at risk of malaria world-wide [1].

Hundreds of millions of ITNs are distributed to households and vulnerable individuals through various mechanisms every year [1]. Consistent and proper use of ITNs, ie, unfurling of ITNs during sleeping hours, requires multiple affirmative behaviors by those at risk of malaria. Access to ITNs is but the first step in a multi-step malaria prevention cascade which begins with ITN access and progresses to ownership, partially effective use, effective use, sustained effective use, and, finally, terminates with malaria prevention (**Figure 1**). While malaria prevention is achieved to varying degrees at each level of effective ITN use, achieving the full potential of ITNs to prevent malaria and sustaining recent achievements in malaria prevention requires not only access to ITNs, but also a renewed commitment to understanding the factors that affect ITN-related behaviors along the entire malaria prevention cascade.

**Figure 1:**
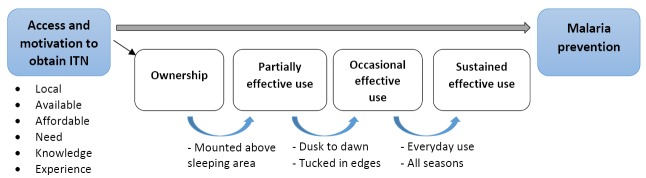
Multi-step cascade for effective malaria prevention with insecticide-treated bednets (ITNs).

Adequate tools for measuring the use of ITNs are essential for assessing the success of interventions to prevent malaria. There is reason to believe that the currently available methods for measuring ITN use are inadequate [4]. To date, there have been no systematic efforts to characterize the comparative accuracy of these methods in assessing ITN adherence. We hypothesize that the commonly used self-report method for measuring ITN use will overestimate ITN use compared to more objective methods. To test this hypothesis and estimate the magnitude of the discrepancy, we undertook this meta-analysis of studies which allow for comparison of self-reported and objectively measured ITN use in malaria endemic settings.

## Conceptual framework

There are a variety of ways to measure adherence to recommended ITN use for the prevention of malaria. A simplistic method is to use ownership of ITNs, with the idea that ownership of ITNs should be valid as a proxy for ITN adherence if individuals value ITNs for the prevention of malaria. This simplistic understanding masks the multiple required ITN-related behaviors in the malaria prevention cascade (**Figure 1**). As has been confirmed in a variety of settings [5-8] it is not possible to merely equate ownership of ITNs with ITN use.

Another low-cost and flexible measure, more feasible than the objective measures listed below for large-scale household surveys, is gathering self-reports of ITN use from individuals or household representatives. Since 2000, Roll Back Malaria has recommended the routine collection of both household possession of ITNs and self-reported ITN use [9]. Self-reports with a short recall window are the most common method: respondents are simply asked about their, or another individuals’, use of an ITN the night before. The vast majority of studies in the peer-reviewed literature assess ITN adherence with self-reported use as the primary, and often exclusive, measure of ITN use [10-14], and very few studies have attempted to examine the reliability of this measure. In the Malaria Indicator Surveys, which are the most common means of monitoring national-level malaria prevention programs to guide public health policy, nets encountered in the household are reviewed with the survey respondent and they are asked whether it was used the night prior and, if so, who slept under that net the last night.

A number of potential biases limit the utility of self-reported measures in accurately and precisely assessing ITN adherence [15]. First, social desirability effects may bias estimates of ITN use upward [16]. This bias may be especially pronounced in settings where there is an injunctive norm favoring ITN use, eg, after large-scale behavior change promotion programs. Even in the setting of partially effective or even ineffective behavior change programs, it is unlikely for social desirability effects to result in downward bias. Second, self-reports could systematically misclassify ITN use due to recall bias [17] from incorrect recollection of adherence the night before by one’s self or others in the household, though one study has shown that recollection for the night before was accurate, while recall accuracy declined over 2-4 weeks [18]. Finally, respondents reporting on the ITN use of others in the household may have incorrect information.

Objective methods have been employed to assess ITN adherence so as to address some of the potential biases in self-reports. Day-time visual observations have been utilized to confirm the presence of a mounted ITN in the household [19-21]. While this method provides an assessment by a more objective observer (and therefore potentially less biased) and confirms whether or not an ITN is mounted above a sleeping area, this method requires entering private spaces and does not assure that anyone actually slept under an unfurled ITN the night before. Furthermore, the ITN may be used during the night and then taken down during the day for cleaning or to secure more space in the living area, thus rendering the day-time observation inaccurate. Unannounced night-time visits have also been used [22,23]. While this method confirms whether someone is actually sleeping under an unfurled ITN, its widespread use is limited by privacy concerns, community acceptability, and logistical challenges. In the context of large-scale population-based surveys (such as the Malaria Indicators Surveys carried out under the Demographic and Health Surveys Program), the cost of such data collection may be prohibitive.

All three of these methods suffer from the important limitation that they assess ITN use at a single moment in time. ITN use during the night is fluid, with individuals coming in and out of the ITN at various times during the night [24], thus self-reported measures (eg, ITN use the prior night, single point-in-time measures of who is under the ITN, or whether an ITN is mounted) cannot accurately characterize time-varying ITN adherence throughout the prior night. In addition, measurements of ITN use on a particular night do not necessarily characterize ITN adherence on other days, as it is well demonstrated that there are variations in ITN use on a nightly, weekly, and seasonal basis [11].

## METHODS

**Figure 2:**
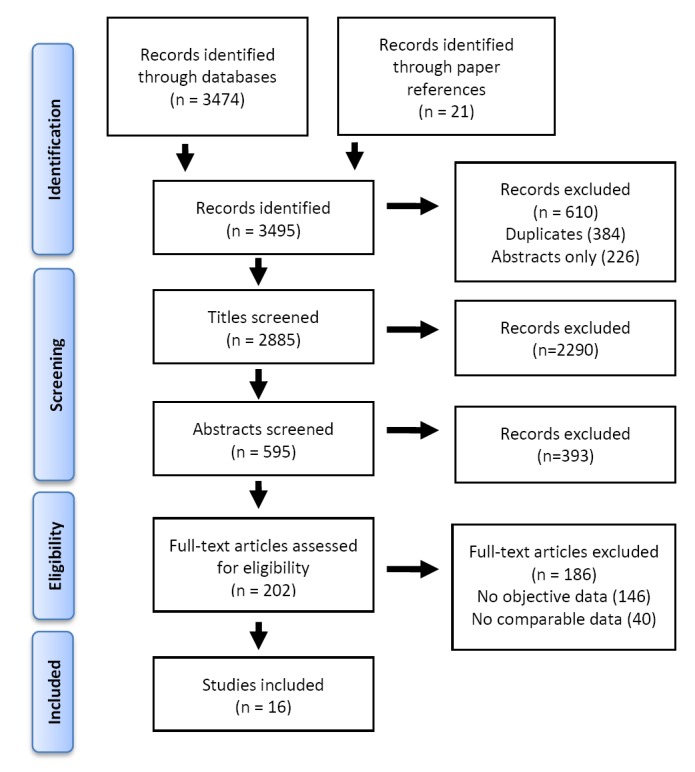
Search strategy (adapted from PRISMA 2009 flowchart).

We conducted a comprehensive search of the literature using the Embase and PubMed electronic databases in November 2015. The searches combined general terms and Medical Subject Headings related to ITNs and malaria (Table S1 in **Online Supplementary Document(Online Supplementary Document)**). Throughout the process we adhered to the PRISMA reporting guidelines for meta-analyses [25] (**Figure 2**). The electronic search was supplemented by scanning the reference lists of included reports. The initial list of 2885 English language, full-text records included 1622 articles from Embase, 1242 from PubMed and 21 from the reference list scan. A preliminary screening step was employed by reviewing the titles of the 2885 articles and removing those not focused on ITN use. The 595 remaining abstracts were then critically reviewed for indications that the full text would provide both a self-reported and an objective measure of ITN use in the same population. The remaining 202 articles were read in full by a single study author (PJK). After applying the inclusion and exclusion criteria described below, 16 articles were included in the meta-analysis and data were extracted using a standardized form.

### Quality assessment

All 16 studies employed a cross-sectional design to assess ITN use and therefore are of “low quality” according to the GRADE system for grading evidence [26]. As the outcome of interest requires the concurrent measurement of ITN use by two separate methods, a quality assessment was performed focusing on areas for potential bias in comparing discrepancies between these measures. Quality measures included the type of study population, use of pre-defined definitions of ITN use, measurement of self-reported and objectively measured ITN use at similar times and among similar subjects and the use of randomized sampling when sampling was undertaken (**Table 2**).

**Table 2:** Studies included in meta-analysis (sorted by date)

	Study	Adherent by SR	Total sample for SR	Percent adherent by SR	Adherent by Obj	Total sample for Obj	Percent adherent by Obj	Percentage point difference (SR-Obj)	Difference relative to Obj	Transmission season of survey	Type of objective measure	Study population	Unit of analysis	Definitions of ITN use		Comparable measures of ITN use with SR and Obj methods		
**Self-report**	**Objective**	**Timing**	**Same subjects**	**Random sampling**
1	Leake et al., Malaysia (1989)	281	548	51.3%	189	494	38.3%	13.0%	34.0%	Low	Night visit; 2100-2400	General	Indiv	Household representative reported	Individual observed under ITN	Obj then SR the following day	Yes	No explanation for missing objective measures
2	Sexton et al., Kenya (1990)	141	166	84.9%	119	166	71.5%	13.3%	18.5%	Holoendemic	Night visit; 2100-2200	Children under 5 years	HH	Households claiming use every night in last 7 days	Using net at night visit (avg two measures 70% and 73%)	SR use every day last 7 nights vs observed use	Yes	N/A
3	Leake et al., Malaysia (1994)	311	676	46.0%	198	537	36.9%	8.8%	23.8%	Low	Night visit; 2100-2400	General	Indiv	Household representative reported	Individual observed under ITN	Obj then SR the following day	Yes	No explanation for missing objective measures
4	Linhua et al., China (1994)	178	226	78.8%	156	226	69.0%	9.7%	14.1%	Unclear	Night visit; 2300	General	HH	Household representative reported	Observed utilization of ITN	Unclear when Obj occurred relative to SR	Yes	N/A
5	Fraser-Hurt et al., Tanzania (1999)	353	360	98.0%	188	190	98.9%	-0.9%	-0.9%	Dry season	Night visit; 0500-0800	Children under 5 years	Indiv	Clinic-reported compliance	Children observed inside ITN	Monthly SR vs surprise Obj	Yes	No explanation for only 229 visits and 17% unavailable
6	Spencer et al., Uganda (2004)	708	1,245	56.9%	682	1,245	54.8%	2.1%	3.8%	Low	Visual inspection during day; 1000-1600	General	Indiv	Household representative reported	Hanging bednet in household of reported use	Concurrent measure of SR and Obj	Yes	N/A
7	Grabowsky et al., Ghana (2005)	168	257	65.4%	170	249	68.3%	-2.9%	-4.3%	High	Visual confirm during day, unclear time	Children under 5 years	Indiv	Household representative reported	Bednet observed hung over child’s bed	Concurrent measure of SR and Obj	Yes	No explanation for missing objective measures
8	Frey et al., Burkina Faso (2006)	177	180	98.3%	142	180	78.9%	19.4%	24.6%	High	Night visit; 2100 or 0500	Children under 5 years	Indiv	Full-time use the previous night	Study child observed under bednet	Obj then SR measure the following day	Yes	N/A
9	Fettene et al., Ethiopia (2009)	77	119	64.7%	60	119	69.9%	14.3%	28.3%	Low	Visual confirm during day	General	HH	Reporting every night use	Households with nets observed hanging	Concurrent measure of SR and Obj	Yes	N/A
10	Cohee et al., Uganda (2009)	123	128	63.1%	10	13	50.4%	19.2%	24.9%	Low	Visual confirm during day	HIV positive patients	HH	At least one person used ITN previous night	Households with nets observed mounted	Unclear when Obj occurred relative to SR	Yes	Randomly selected households for observations
11	Becker-Dreps et al., DRC (2009)	87	103	84.4%	72	103	70.0%	14.4%	20.6%	Unclear	Visual confirm during day	Pregnant women	Indiv	“[E]very day or almost every day”	ITNs hanging “in the correct position”	Concurrent measure of SR and Obj	Yes	N/A
12	Gobena et al., Ethiopia (2010)	630	1,879	33.5%	392	1,879	20.9%	12.7%	60.7%	High	Visual confirm during day	General	HH	Household reported use the night before	ITN hung “above bed or sleeping place”	Concurrent measure of SR and Obj	Yes	N/A
13	Macintyre et al., Zambia (2011)	**271**	**483**	**56.1%**	**283**	**483**	**58.6%**	**-2.5%**	**-4.3%**	High	Visual confirm during day	Children under 5 years	HH	At least one person used ITN previous night	At least one ITN observed hanging	Concurrent measure of SR and Obj	Yes	N/A
14	Dori et al., Ethiopia (2012)	367	609	60.3%	228	609	37.4%	22.8%	61.0%	High	Visual confirm during day	General	HH	Report currently “using a net”	Observed hung from “ceiling over a bed”	Concurrent measure of SR and Obj	Yes	N/A
15	C-Change et al., Ethiopia (2012)	**88**	**273**	**32.3%**	**94**	**273**	**34.3%**	**-2.0%**	**-6.2%**	Low	Visual confirm during day	General	HH	Someone slept under bednet previous night	At least one hanging “from wall or ceiling”	Concurrent measure of SR and Obj	Yes	N/A
16	Deressa et al., Ethiopia (2014)	524	755	69.4%	567	755	75.1%	-5.7%	-7.6%	High	Visual confirm during day	General	HH	Someone slept under bednet previous night	Households with bednet hung over bed	Concurrent measure of SR and Obj	Yes	N/A

SR – self-reported measure, Obj – objective measure, Indiv – individual, HH – Household, ITN – insecticide-treated net, N/A – not applicable

One article, in Burkina Faso [24], provided two eligible sets of measurements, one each in the dry and wet seasons. The results from the wet season were used in the meta-analysis because the discrepancy was smaller during that time window. Two studies did not clearly specify when the study occurred in relation to the malaria transmission season. There were a range of different definitions for ITN use, especially in relation to the objective measure. In general, the night visits counted people who were under unfurled ITNs. However, the definition of visual confirmation of ITNs mounted in the household ranged from ITNs hung “above bed or sleeping place” [27] to the more specific observation of an ITN observed hanging over a subject child’s bed [21]. Most objective measures using visual confirmation occurred concurrently with the self-reported use, while the night visits were commonly paired with self-reported use measurements the following day. Two studies were unclear about when the visual inspections occurred relative to the self-reports [22,28] and another study reported average self-reported ITN use the night prior from multiple measures (monthly) and a single night visit towards the end of the study [29]. All studies assessed self-reported and objectively measured ITN use among the same group of study participants, however, four studies had unexplained larger samples for the self-reports than objective measures [21,25,30]. Although not specified in the studies, these different sample sizes may be due to logistical challenges implementing the objective measures, participant refusal or loss to follow up. This differential response on the objective measure could bias the objective measures towards higher apparent ITN use and potentially increase the overestimation between self-reports and objective measures. A sensitivity analysis excluding these four studies, however, showed a one percentage point increase in the overestimation due to self-reports, so these studies were retained as a conservative measure. One study used a random sample of households from the self-reported group for the objective measure [22]. Overall, the potential for biases due to comparing cross-sectional studies and different definitions of ITN use was managed with strict inclusion criteria for comparable measures of self-reported and objectively measured ITN use as detailed below.

### Definition of comparable measures

In order to be included in the meta-analysis, studies were required to contain comparable data on self-reported and objectively measured ITN use. The data were considered “comparable” when there was an objective measure of ITN use at the same unit of analysis, covering the same time period and obtained in the same population as a self-reported measure of ITN use. For example, we included studies that elicited self-reported ITN use and then confirmed ITN use with unannounced night visits in the same households. We excluded studies with discrepant units of analysis (eg, ITN use was objectively measured at the household level but was based on self-report at the individual level) or discrepant time windows (eg, objective measure of ITN use was obtained in one season while self-report was elicited in another season). Objective measures were categorized as either: 1) unannounced night visits confirming ITN use at the time of the visit or 2) visual confirmation of a mounted ITN (**Table 1**). We also collected data on the year of publication, country, sample size, unit of analysis, season of study and type of objective measure utilized.

**Table 1:** Definition of comparable self-reported and objectively measured ITN use for inclusion

	Objectively measured ITN use	
**Self-reported ITN use**	**Unannounced home visits confirming**	**Visual confirmation of mounted ITN over:**
Individual level	The individual’s use of the ITN	The individual’s sleeping area
Household level	At least one ITN in use in household	At least one sleeping area in household

ITN – insecticide-treated net

### Statistical analyses

All statistical analyses were performed in Stata 10 using the *metan* command and related modules (StataCorpCollege Station, TX, USA). In the meta-analysis, a random effects model was used to determine a weighted average risk difference, with 95% confidence intervals (CI), between self-reported ITN use and objectively measured ITN use. This discrepancy between self-reported (SR) and objectively measured (Obj) ITN use was then compared to a weighted average of objectively measured ITN use using the *metaprop* command for meta-analyses of proportions [31].

**Equation 1.** (Use_SR_ – Use_Obj_) / Use_Obj_ = Over- or under-estimation Use_SR_ relative to Use_Obj_

Heterogeneity was assessed using the I-squared statistic. Individual studies were assessed for influence on the final result by omitting each study and re-estimating the pooled estimate using the *metaninf* command [32]. A L’Abbe plot was used to explore potential causes of heterogeneity due to event rates in the two groups.

Additional stratified analyses were conducted to explore the heterogeneity. First, we compared studies with the year of publication before and after the signing of the Abuja Declaration in 2000, to explore differences due to increasing prominence of ITNs in malaria prevention. Second, we compared unannounced night visits to visual confirmation as the objective measure reasoning that visual confirmation of mounted ITNs may not be as accurate as directly observed ITN use during night visits. Third, we compared studies based on the season of assessment, high vs low transmission, as defined in the paper. Fourth, we compared studies based on the type of study population, defined as general population vs specialized population (pregnant women, children under five years, HIV positive). Finally, we compared studies according to the unit of analysis.

### Role of the funding source

The funding source had no role in the writing of the manuscript nor in the decision to submit it for publication.

## RESULTS

### Study characteristics

Sixteen articles, published between 1989 and 2014, were selected for inclusion [15,19,22,24,27-38] (**Table 2**). Five studies were published before 2000 (31%). Six studies (38%) used unannounced night visits as the objective measure. The unit of analysis was at the individual level in 7 studies (43.8%). Self-reported ITN use ranged from 32.3% [37] to 98.3% [24], with an unweighted average of 67.3% (95% CI: 55.9% to 78.7%). Objectively measured ITN use ranged from 20.9% to 98.9% [30], with an unweighted average of 58.8% (95% CI: 47.6% to 69.9%).

### Main results

**Figure 3:**
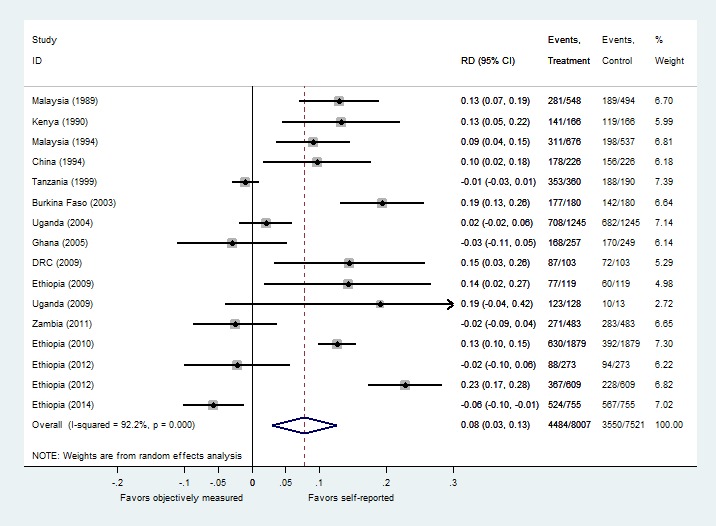
Meta-analysis of discrepancies between self-reported and objectively measured insecticide-treated bednets (ITN) use.

In 11 studies (69%), the rate of self-reported ITN use exceeded the rate of objectively measured ITN use. The discrepancy between the self-report and objective measures ranged from 22.8% in favor of self-report in Ethiopia [38] to 5.7% in favor of objective measurement in another study from Ethiopia [39]. Across all studies, self-reported ITN use was 8 percentage points (95% CI: 3 to 13) higher than objectively measured ITN use (**Figure 3**). This non-stratified analysis demonstrated substantial heterogeneity with an I-squared statistic of 92%. The weighted average rate of self-reported ITN use was 67% (95% CI: 54% to 81%). The weighted average rate of objectively measured ITN use 59% (95% CI: 42% to 75%). Using the weighted average of the objective measures (Obj) as a reference for ITN adherence, self-reported measures (SR) overestimated the rate of ITN use relative to objectively measured ITN use by Eq. 1: (67 – 59) / 59 = 8 / 59% = 13.6%.

### Supplementary analyses

According to the influence analysis, no single study drove the main result (Figure S1 in **Online Supplementary Document(Online Supplementary Document)**). No systematic difference in the discrepancies was discernible in the L’Abbe plot based on the levels of adherence captured by the two methods (Figure S2 in **Online Supplementary Document(Online Supplementary Document)**).

**Figure 4:**
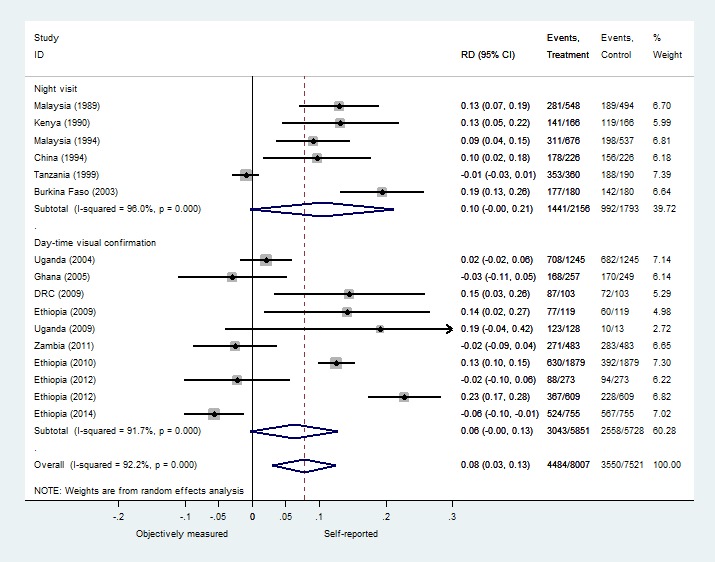
Stratified meta-analysis of discrepancies between self-reported and objectively measured insecticide-treated bednets (ITN) use by type of objective measure.

In general, none of the stratified estimates yielded significantly improved measures of heterogeneity, nor did any yield statistically significant differences in pooled estimates between the groups; all I-squared values were 71% or higher. Stratifying the studies pre- and post-Abuja, the unit of analysis and the season of study identified no significant difference in the pooled estimates (Figures S3, S4 and S5 in **Online Supplementary Document(Online Supplementary Document)**). The studies using unannounced night visits had a 10.5% discrepancy favoring the self-reported measures compared to 6.3% when using the visual confirmation method, but the comparison was not statistically significant (**Figure 4**). General study populations demonstrated more discrepancy in favor of self-reported use than studies measuring ITN use in special populations, but this difference was not statistically significant (10.3% vs 3.2% respectively) (Figure S6 in **Online Supplementary Document(Online Supplementary Document)**).

## DISCUSSION

This meta-analysis comparing studies that report comparable self-reported and objectively measured ITN use in the same population found that self-reports overestimate ITN adherence. Average self-reported ITN use was 67% compared to 59% for objectively measured ITN use across the 16 studies. Thus, self-reported use overestimated actual use by an average of 8 percentage points or, relative to the objective measurements, by (67 – 59) / 59 = 13.6%. This is the first meta-analysis to estimate the comparative accuracy of methods for assessing adherence to ITN use. The magnitude of this finding is in line with other systematic reviews showing a 10%-20% overestimate in self-reported adherence to HIV medications confirmed with electronic monitoring [39,40].

One study not included in this meta-analysis used an innovative electronic objective monitoring device to assess the state of the ITN and also performed self-reported measures. This study found no significant difference between the objective monitor measurements and self-reports of use when individuals were asked the next morning. The reason it was not included in the meta-analysis, however, is that the participants were aware that their ITN use was being tracked by an objective monitor. These self-reports, then, are potentially biased upward in the same way that self-reported ITN use would be upward biased if participants reported their ITN use the morning after survey assessors had just performed a surprise night visit. Electronic monitoring tools may be able to help assess social desirability bias in ITN use self-reports if the self-reports are measured in a context distinct from the objective monitoring, eg, in a clinic by different personnel.

Systematic overestimation inherent in self-reported measures of ITN use may not be surprising given the nature of self-reports of health behaviors [16]. However, we found substantial variation in the overestimation between studies which provides further evidence of the unreliability of self-reports for assessing ITN use. First, there was significant variation in the magnitude of overestimation when relying on self-reported ITN use, ranging from 61% [35] to -7.6% underestimation [39]. Second, there was sizeable heterogeneity between the studies that was not explained by the available study-level covariates. The wide variance in the magnitude of the overestimation and the inability to predict, even within the same country, the degree to which self-reported measures overestimate ITN use, suggest that single measures of self-reported ITN use are a problematic tool for assessing the actual rate of ITN adherence in a population.

This finding has important implications for malaria prevention programs. First, since the majority of data about ITN adherence is based upon self-reported use, policy makers are potentially relying on inaccurate information when they make decisions about the optimal deployment of ITNs. Second, the systematic upward bias in self-reported rates of ITN use implies that ITNs may have greater efficacy than previously thought. A recent study found that ITNs accounted for 68% of a 40% decline in clinical incidence of malaria in Africa from 2000 to 2015 [41]. Similarly, a case-control study found that mass distributing ITNs in Benin provided a 41%-55% protection against clinical malaria cases among children under 5 years of age [42]. Both of these studies base their estimates of ITN efficacy on self-reported rates of ITN use. If self-reported use is indeed overestimated, as our results suggest, then the actual efficacy of ITNs may be significantly higher and the reported cost-effectiveness of ITNs may be undervalued. Third, our findings suggest that evaluations of ITN use promotion interventions (eg, behavior change campaigns) and attempts to model the optimal mix of malaria prevention measures should use caution in relying on self-reported ITN use measures.

To address these challenges, studies should recognize the identified limitations of self-reported ITN use in interpreting and discussing ITN adherence using that measure. Self-reported measures may provide a “rough” estimate of ITN use, but they may be less useful as estimates of actual ITN adherence. This study’s findings show there was substantial unexplained heterogeneity detected in the discrepancy between self-reported and objective measured ITN use. This heterogeneity makes it difficult to conclude, in any particular case, that self-reports are overestimating ITN use by a specific amount, but the magnitude, statistical significance and consistency of the overestimation when relying on self-reports all suggest that self-reported ITN use should be interpreted with caution.

Another possible solution could be efforts to utilize more objective methods in assessments of ITN adherence. Due to cost and time constraints, unannounced night visits and visual confirmation may not be feasible in many situations, although using these objective measures on a smaller scale to validate self-reported estimates of use may be more feasible. New measurement tools are being developed, such as electronic ITN adherence monitors [18,43,44], which may reduce some of the practical problems with current objective methods and allow for easier objective and longitudinal assessment of ITN adherence. While it is likely that self-reported ITN use will continue to be the most feasible tool for assessing ITN use in large-scale household surveys, newer electronic adherence monitoring tools may add value in small-scale observational studies and randomized trials where accurately assessing ITN adherence is more crucial.

Interpretation of our findings is subject to several limitations. We were unable to identify study-level variables to explain the high level of heterogeneity, though such heterogeneity is common in the ITN use literature [45]. After a broad search, we still may have missed some studies. Out of more than 2000 records screened, we were able to identify only 16 studies for inclusion. Furthermore, we excluded some studies that performed objective measurements if the data could not be compared in a valid manner to self-reports. It was not always clear from the reported methods whether the self-reported and objective measures were obtained at the same time and studies obtaining self-reported and objective measures on different days could have introduced additional heterogeneity. There is no accepted gold-standard for measuring bednet adherence and even the objective measures used here have limitations. Using the visualization of a hanging ITN over a sleeping area as the metric for ITN use may bias ITN adherence up if a mounted ITN was not actually used the night before, or may bias ITN adherence down if an ITN that was used the night before was then taken down for cleaning during the assessment. These distinctions have important programmatic implications which are not fully explored in this study, but have been elsewhere [46]. Unannounced night visits may not find someone under an ITN at the time of the visit, even if they end up using the ITN that night. Of the six studies which used night visits, most started at 9PM, and some checked as late as 8AM, which may be too early or too late to capture people who actually used an ITN during that night. In those cases self-reported use may still be accurate even if the night visit did not find individuals under the ITN, and the apparent discrepancy between the two would be smaller. Finally, none of the studies were specifically designed to estimate the discrepancy between self-reported and objectively measured ITN use, thus the results of this study, especially given the significant heterogeneity, are best interpreted in general terms and not likely to be an exact estimate.

## CONCLUSIONS

In this meta-analysis, studies that reported ITN use using both self-reports and at least one other objective measure demonstrated an over-estimation due to self-reports; self-reported ITN use was 8 percentage points higher, representing a 13.6% overestimation relative to objectively measured ITN use. These findings raise questions about the reliability of using self-reported ITN use alone as a surveillance tool and a guide for making policy decisions.
