# Supplementary material for: Quantifying bias in measuring insecticide-treated bednet use: meta-analysis of self-reported vs objectively measured adherence
Source: J Glob Health. 2018 Mar 31;8(1):010411. doi: 10.7189/jogh.08.010411 (PMC5878861; doi:10.7189/jogh.08.010411)
Supplement: Online Supplementary Document [file jogh-08-010411-s001.pdf]

Online Supplementary Document

Krezanoski et al. Quantifying bias in measuring insecticide-treated bednet use: meta-analysis of self-reported vs objectively measured adherence

J Glob Health 2018;8:010411

Table S1

Search terms

Pubmed:

("theory"[TIAB] OR "theoretical"[TIAB] OR "Health Services Needs and Demand"[MeSH Terms] OR "Behavior"[MeSH Terms] OR "use"[TIAB] OR "using"[TIAB] OR "uses"[TIAB] OR "usage"[TIAB] OR "non-use"[TIAB] OR "utilize"[TIAB] OR "utilizes"[TIAB] OR "utilization"[TIAB] OR "non-utilization"[TIAB] OR"hang"[TIAB] OR "hanging"[TIAB] OR "compliance"[TIAB] OR "non-compliance"[TIAB] OR"comply"[TIAB] OR"adhere"[TIAB] OR"adherence"[TIAB] OR "non-adherence"[TIAB] OR "uptake"[TIAB] OR "ownership"[TIAB] OR "own"[TIAB] OR "owning"[TIAB] OR "possession"[TIAB] OR "mount"[TIAB] OR "mounting"[TIAB] OR "coverage"[TIAB] OR "attrition"[TIAB] OR "persistence"[TIAB] OR "behaviors"[TIAB] OR "behavior"[TIAB] OR "motivation"[TIAB] OR "attitudes"[TIAB] OR "acquire"[TIAB] OR "obtain"[TIAB] OR "unfurl"[TIAB] OR "furl"[TIAB] OR "buy"[TIAB] OR "purchase"[TIAB] OR "seek"[TIAB] OR "demand"[TIAB] OR "demands"[TIAB] OR "demanding"[TIAB]) AND ("long-lasting insecticidal"[TIAB] OR "insecticide treated"[TIAB] OR "LLIN"[TIAB] OR "LLINs"[TIAB] OR "ITN"[TIAB] OR "ITNs"[TIAB] OR "bednet"[TIAB] OR "bednets"[TIAB] OR "bed net"[TIAB] OR "bed nets"[TIAB] OR "mosquito net"[TIAB] OR "mosquito nets"[TIAB] OR "netting"[TIAB] OR "Mosquito Nets"[MeSH Terms] OR "Insecticide-Treated Bednets"[MeSH Terms]) AND ("malaria"[MeSH Terms] OR "malaria"[TIAB])

Embase:

'malaria':ti,ab OR 'malaria':de AND ('long-lasting insecticidal':ti,ab OR 'insecticide treated':ti,ab OR 'llin':ti,ab OR 'llins':ti,ab OR 'itn':ti,ab OR 'itns':ti,ab OR 'bednet':ti,ab OR 'bednets':ti,ab OR 'bed net':ti,ab OR 'bed nets':ti,ab OR 'mosquito net':ti,ab OR 'mosquito nets':ti,ab OR 'netting':ti,ab) AND ('theory':ti,ab OR 'theoretical':ti,ab OR 'use':ti,ab OR 'using':ti,ab OR 'uses':ti,ab OR 'usage':ti,ab OR 'non-use':ti,ab OR 'utilize':ti,ab OR 'utilizes':ti,ab OR 'utilization':ti,ab OR 'non-utilization':ti,ab OR 'hang':ti,ab OR 'hanging':ti,ab OR 'compliance':ti,ab OR 'non-compliance':ti,ab OR 'comply':ti,ab OR 'adhere':ti,ab OR 'adherence':ti,ab OR 'non-adherence':ti,ab OR 'uptake':ti,ab OR 'ownership':ti,ab OR 'own':ti,ab OR 'owning':ti,ab OR 'possession':ti,ab OR 'mount':ti,ab OR 'mounting':ti,ab OR 'coverage':ti,ab OR 'attrition':ti,ab OR 'persistence':ti,ab OR 'behaviors':ti,ab OR 'behavior':ti,ab OR 'motivation':ti,ab OR 'attitudes':ti,ab OR 'acquire':ti,ab OR 'obtain':ti,ab OR 'unfurl':ti,ab OR 'furl':ti,ab OR 'buy':ti,ab OR 'purchase':ti,ab OR 'seek':ti,ab OR 'demand':ti,ab OR 'demands':ti,ab OR 'demanding':ti,ab) AND [embase]/lim

Figure S1

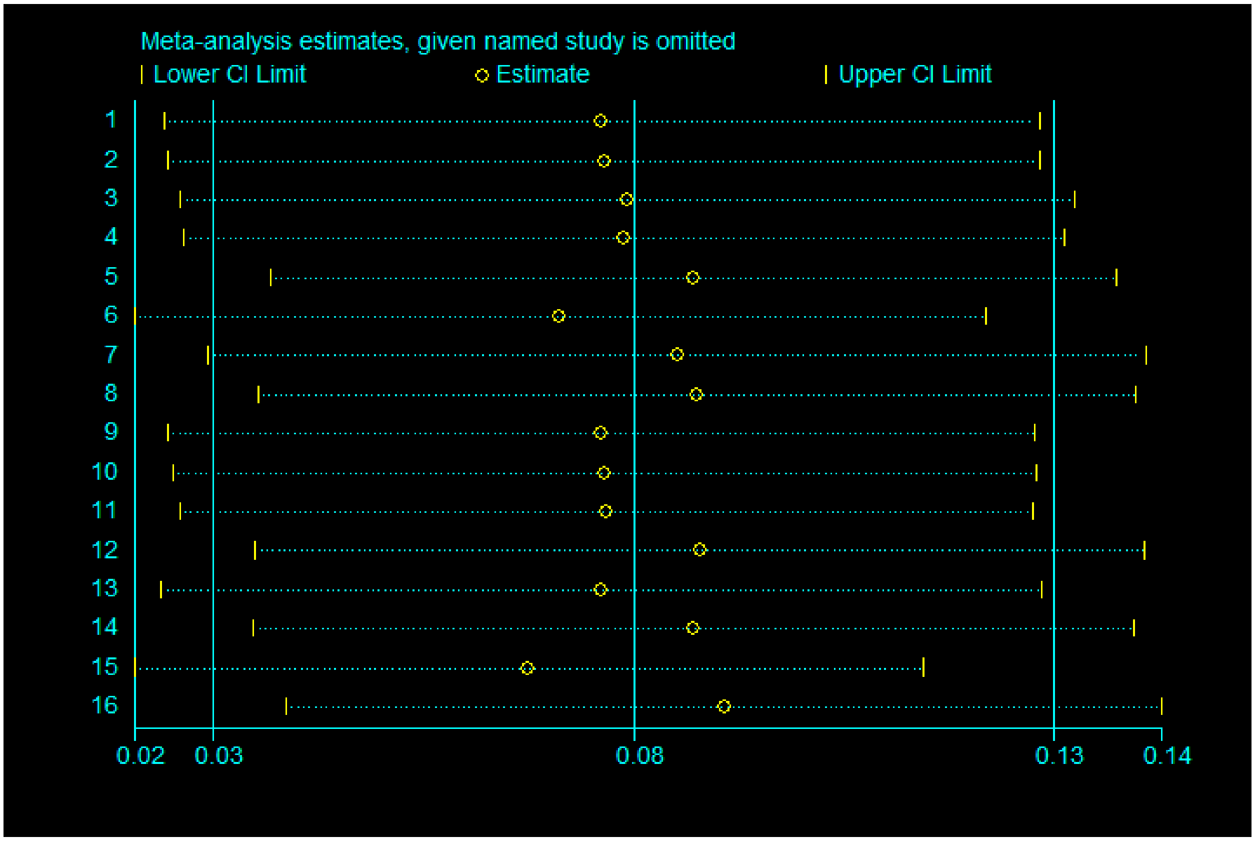
Meta-analysis estimates, given named study is omitted
Lower CI Limit
Estimate
Upper CI Limit
1
2
3
4
5
6
7
8
9
10
11
12
13
14
15
16
0.02
0.03
0.08
0.13
0.14

Figure S2

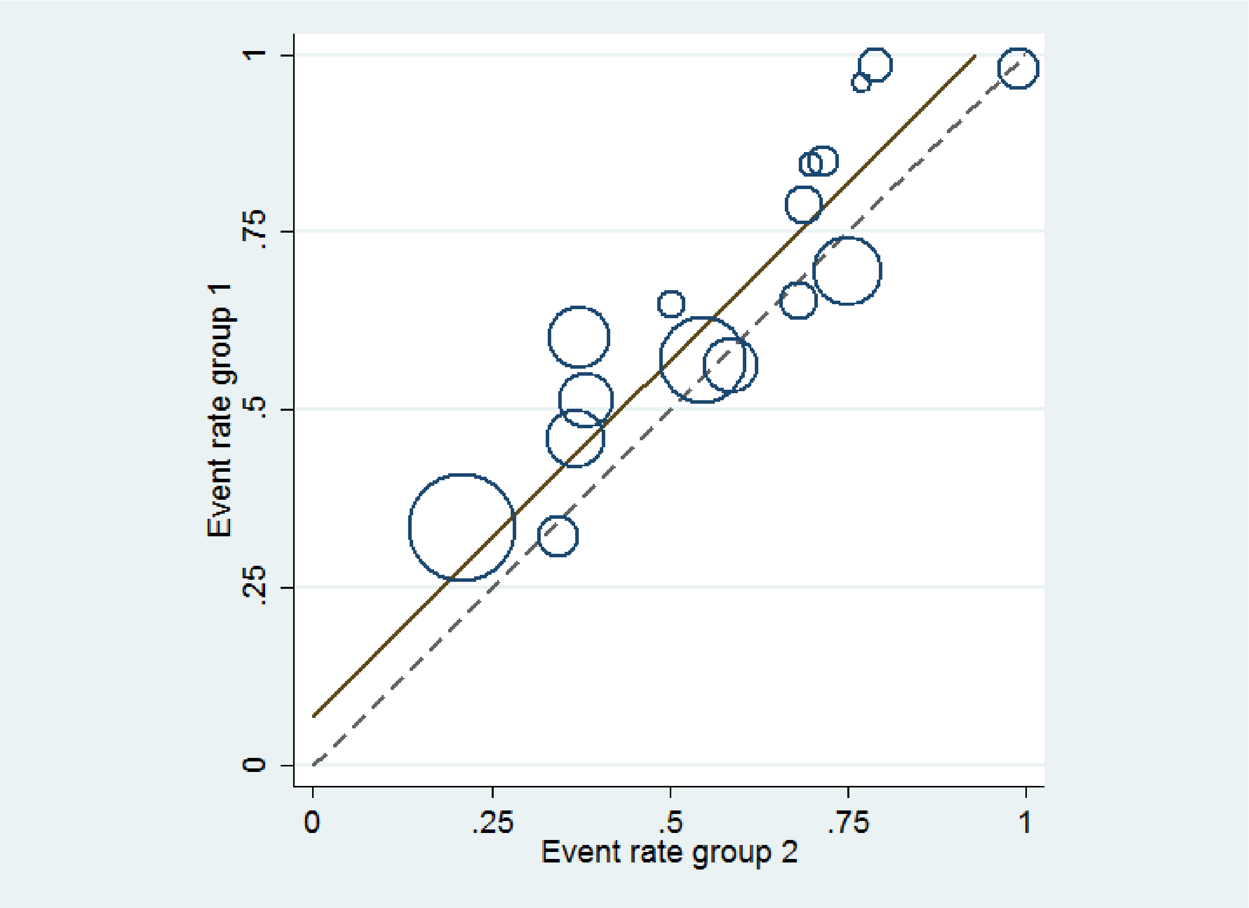

Figure S3

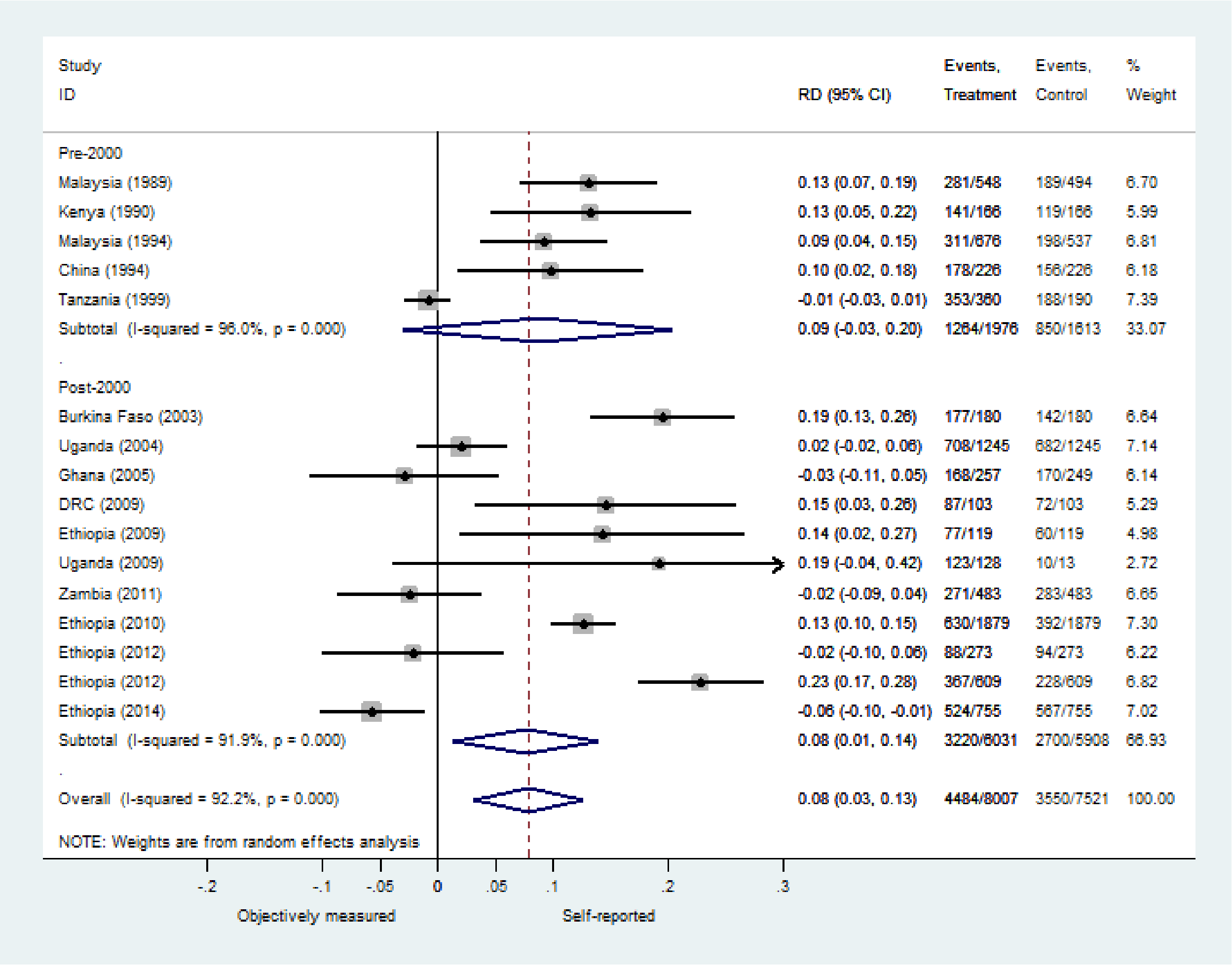
Study
ID
RD (95% CI)
Events,
Treatment
Events,
Control
%
Weight
Pre-2000
Malaysia (1989) 0.13 (0.07, 0.19) 281/548 189/494 6.70
Kenya (1990) 0.13 (0.05, 0.22) 141/166 119/166 5.99
Malaysia (1994) 0.09 (0.04, 0.15) 311/676 198/537 6.81
China (1994) 0.10 (0.02, 0.18) 178/226 156/226 6.18
Tanzania (1999) -0.01 (-0.03, 0.01) 353/360 188/190 7.39
Subtotal (I-squared = 96.0%, p = 0.000) 0.09 (-0.03, 0.20) 1264/1976 850/1613 33.07
Post-2000
Burkina Faso (2003) 0.19 (0.13, 0.26) 177/180 142/180 6.64
Uganda (2004) 0.02 (-0.02, 0.06) 708/1245 682/1245 7.14
Ghana (2005) -0.03 (-0.11, 0.05) 168/257 170/249 6.14
DRC (2009) 0.15 (0.03, 0.26) 87/103 72/103 5.29
Ethiopia (2009) 0.14 (0.02, 0.27) 77/119 60/119 4.98
Uganda (2009) 0.19 (-0.04, 0.42) 123/128 10/13 2.72
Zambia (2011) -0.02 (-0.09, 0.04) 271/483 283/483 6.65
Ethiopia (2010) 0.13 (0.10, 0.15) 630/1879 392/1879 7.30
Ethiopia (2012) -0.02 (-0.10, 0.06) 88/273 94/273 6.22
Ethiopia (2012) 0.23 (0.17, 0.28) 367/609 228/609 6.82
Ethiopia (2014) -0.06 (-0.10, -0.01) 524/755 567/755 7.02
Subtotal (I-squared = 91.9%, p = 0.000) 0.08 (0.01, 0.14) 3220/6031 2700/5908 66.93
Overall (I-squared = 92.2%, p = 0.000) 0.08 (0.03, 0.13) 4484/8007 3550/7521 100.00
NOTE: Weights are from random effects analysis
-.2 -.1 -.05 0 .05 .1 .2 .3
Objectively measured
Self-reported

Figure S4

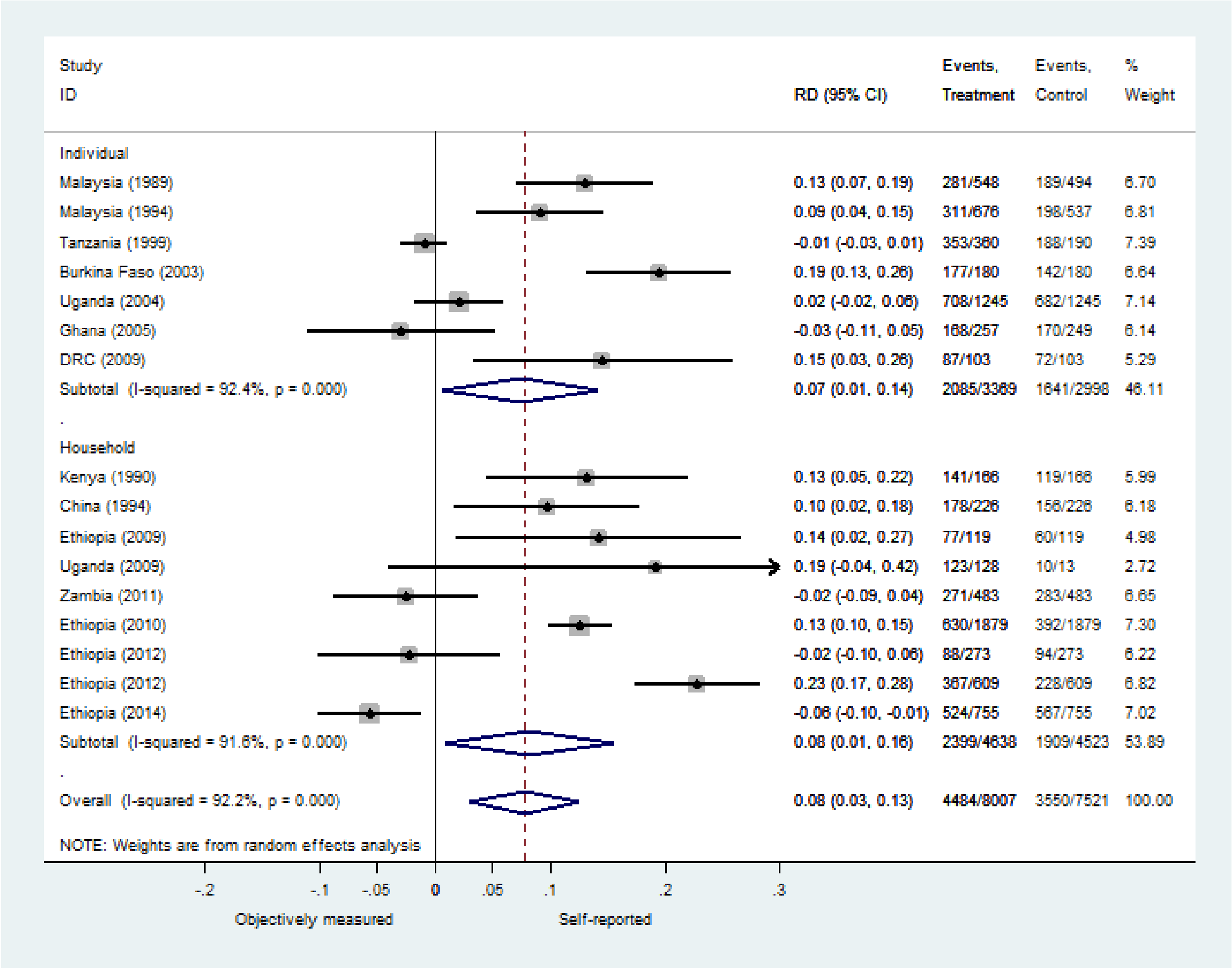
Study
ID
RD (95% CI)
Events, Treatment
Events, Control
% Weight
Individual
Malaysia (1989) 0.13 (0.07, 0.19) 281/548 189/494 6.70
Malaysia (1994) 0.09 (0.04, 0.15) 311/676 198/537 6.81
Tanzania (1999) -0.01 (-0.03, 0.01) 353/360 188/190 7.39
Burkina Faso (2003) 0.19 (0.13, 0.26) 177/180 142/180 6.64
Uganda (2004) 0.02 (-0.02, 0.06) 708/1245 682/1245 7.14
Ghana (2005) -0.03 (-0.11, 0.05) 168/257 170/249 6.14
DRC (2009) 0.15 (0.03, 0.26) 87/103 72/103 5.29
Subtotal (I-squared = 92.4%, p = 0.000) 0.07 (0.01, 0.14) 2085/3369 1641/2998 46.11
.
Household
Kenya (1990) 0.13 (0.05, 0.22) 141/166 119/166 5.99
China (1994) 0.10 (0.02, 0.18) 178/226 156/226 6.18
Ethiopia (2009) 0.14 (0.02, 0.27) 77/119 60/119 4.98
Uganda (2009) 0.19 (-0.04, 0.42) 123/128 10/13 2.72
Zambia (2011) -0.02 (-0.09, 0.04) 271/483 283/483 6.65
Ethiopia (2010) 0.13 (0.10, 0.15) 630/1879 392/1879 7.30
Ethiopia (2012) -0.02 (-0.10, 0.06) 88/273 94/273 6.22
Ethiopia (2012) 0.23 (0.17, 0.28) 367/609 228/609 6.82
Ethiopia (2014) -0.06 (-0.10, -0.01) 524/755 567/755 7.02
Subtotal (I-squared = 91.6%, p = 0.000) 0.08 (0.01, 0.16) 2399/4638 1909/4523 53.89
.
Overall (I-squared = 92.2%, p = 0.000) 0.08 (0.03, 0.13) 4484/8007 3550/7521 100.00
NOTE: Weights are from random effects analysis
-.2
-.1
-.05
0
.05
.1
.2
.3
Objectively measured
Self-reported

Figure S5

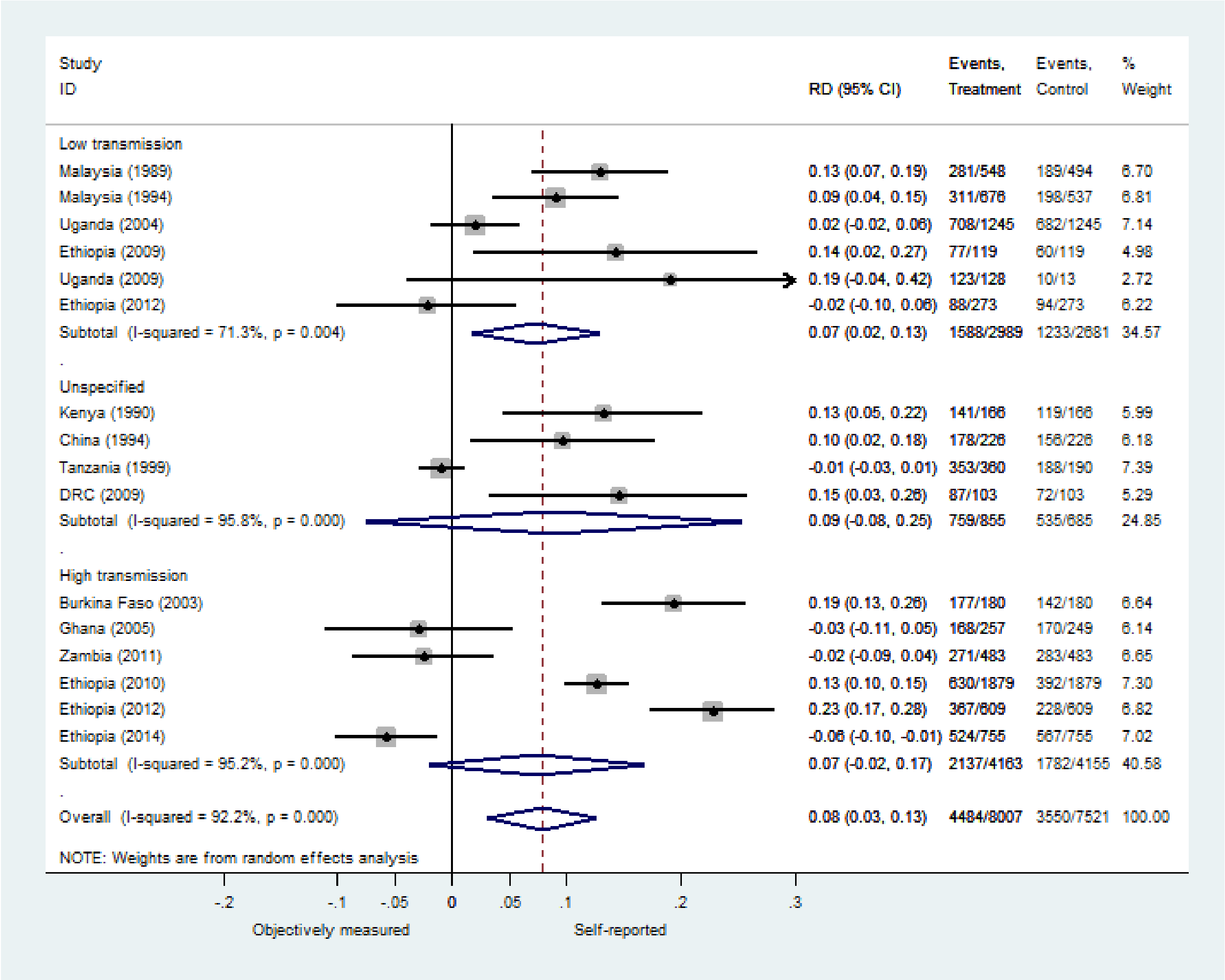
Study ID
RD (95% CI)
Events, Treatment
Events, Control
% Weight
Low transmission
Malaysia (1989) 0.13 (0.07, 0.19) 281/548 189/494 6.70
Malaysia (1994) 0.09 (0.04, 0.15) 311/676 198/537 6.81
Uganda (2004) 0.02 (-0.02, 0.06) 708/1245 682/1245 7.14
Ethiopia (2009) 0.14 (0.02, 0.27) 77/119 60/119 4.98
Uganda (2009) 0.19 (-0.04, 0.42) 123/128 10/13 2.72
Ethiopia (2012) -0.02 (-0.10, 0.06) 88/273 94/273 6.22
Subtotal (I-squared = 71.3%, p = 0.004) 0.07 (0.02, 0.13) 1588/2989 1233/2681 34.57
Unspecified
Kenya (1990) 0.13 (0.05, 0.22) 141/166 119/166 5.99
China (1994) 0.10 (0.02, 0.18) 178/226 156/226 6.18
Tanzania (1999) -0.01 (-0.03, 0.01) 353/360 188/190 7.39
DRC (2009) 0.15 (0.03, 0.26) 87/103 72/103 5.29
Subtotal (I-squared = 95.8%, p = 0.000) 0.09 (-0.08, 0.25) 759/855 535/685 24.85
High transmission
Burkina Faso (2003) 0.19 (0.13, 0.26) 177/180 142/180 6.64
Ghana (2005) -0.03 (-0.11, 0.05) 168/257 170/249 6.14
Zambia (2011) -0.02 (-0.09, 0.04) 271/483 283/483 6.65
Ethiopia (2010) 0.13 (0.10, 0.15) 630/1879 392/1879 7.30
Ethiopia (2012) 0.23 (0.17, 0.28) 367/609 228/609 6.82
Ethiopia (2014) -0.06 (-0.10, -0.01) 524/755 567/755 7.02
Subtotal (I-squared = 95.2%, p = 0.000) 0.07 (-0.02, 0.17) 2137/4163 1782/4155 40.58
Overall (I-squared = 92.2%, p = 0.000) 0.08 (0.03, 0.13) 4484/8007 3550/7521 100.00
NOTE: Weights are from random effects analysis
-.2
-.1
-.05
0
.05
.1
.2
.3
Objectively measured
Self-reported

Figure S6

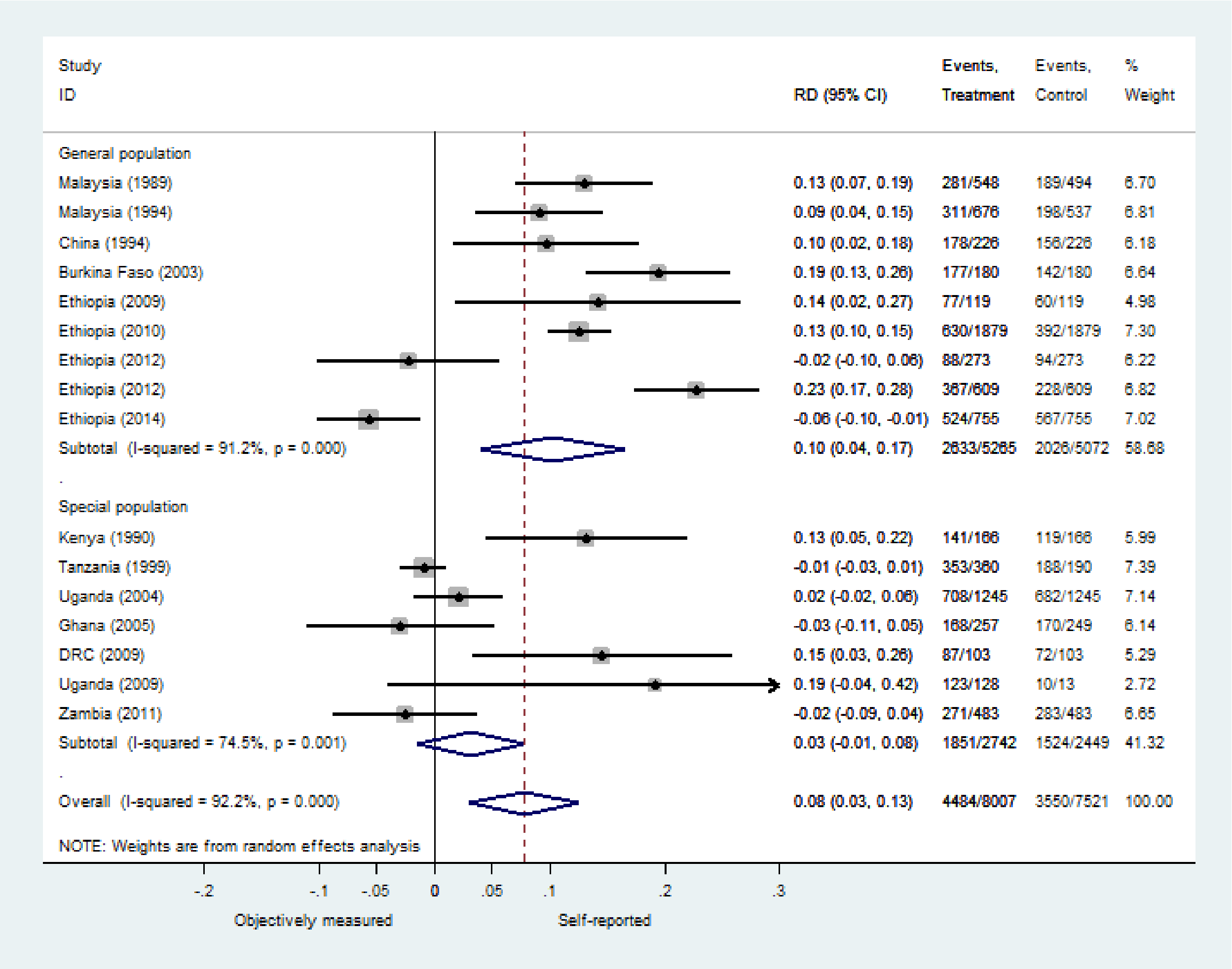
Study
ID
RD (95% CI)
Events, Treatment
Events, Control
% Weight
General population
Malaysia (1989) 0.13 (0.07, 0.19) 281/548 189/494 6.70
Malaysia (1994) 0.09 (0.04, 0.15) 311/676 198/537 6.81
China (1994) 0.10 (0.02, 0.18) 178/226 156/226 6.18
Burkina Faso (2003) 0.19 (0.13, 0.26) 177/180 142/180 6.64
Ethiopia (2009) 0.14 (0.02, 0.27) 77/119 60/119 4.98
Ethiopia (2010) 0.13 (0.10, 0.15) 630/1879 392/1879 7.30
Ethiopia (2012) -0.02 (-0.10, 0.06) 88/273 94/273 6.22
Ethiopia (2012) 0.23 (0.17, 0.28) 367/609 228/609 6.82
Ethiopia (2014) -0.06 (-0.10, -0.01) 524/755 567/755 7.02
Subtotal (I-squared = 91.2%, p = 0.000) 0.10 (0.04, 0.17) 2633/5265 2026/5072 58.68
.
Special population
Kenya (1990) 0.13 (0.05, 0.22) 141/166 119/166 5.99
Tanzania (1999) -0.01 (-0.03, 0.01) 353/360 188/190 7.39
Uganda (2004) 0.02 (-0.02, 0.06) 708/1245 682/1245 7.14
Ghana (2005) -0.03 (-0.11, 0.05) 168/257 170/249 6.14
DRC (2009) 0.15 (0.03, 0.26) 87/103 72/103 5.29
Uganda (2009) 0.19 (-0.04, 0.42) 123/128 10/13 2.72
Zambia (2011) -0.02 (-0.09, 0.04) 271/483 283/483 6.65
Subtotal (I-squared = 74.5%, p = 0.001) 0.03 (-0.01, 0.08) 1851/2742 1524/2449 41.32
.
Overall (I-squared = 92.2%, p = 0.000) 0.08 (0.03, 0.13) 4484/8007 3550/7521 100.00
NOTE: Weights are from random effects analysis
-.2
-.1
-.05
0
.05
.1
.2
.3
Objectively measured
Self-reported
